# Rate of progression of CT-quantified emphysema in male current and ex-smokers: a follow-up study

**DOI:** 10.1186/1465-9921-14-55

**Published:** 2013-05-20

**Authors:** Firdaus AA Mohamed Hoesein, Pieter Zanen, Pim A de Jong, Bram van Ginneken, H Marike Boezen, Harry JM Groen, Mathijs Oudkerk, Harry J de Koning, Dirkje S Postma, Jan-Willem J Lammers

**Affiliations:** 1 Department of Respiratory Medicine, Division of Heart & Lungs, University Medical Center Utrecht, PO box 85500, Utrecht 3508 GA, the Netherlands; 2 Department of Radiology, University Medical Center Utrecht, Heidelberglaan 100, PO box 85500, Utrecht 3508 GA, the Netherlands; 3 Department of Radiology, Radboud University Nijmegen Medical Center, PO box 9101, Nijmegen 6500 HB, the Netherlands; 4 Department of Epidemiology, University Medical Center Groningen, University of Groningen, PO box 30001, Groningen 9700 RB, the Netherlands; 5 Department of Pulmonology, University Medical Center Groningen, University of Groningen, PO box 30001, Groningen 9700 RB, the Netherlands; 6 Department of Radiology, University Medical Center Groningen, University of Groningen, PO box 30001, Groningen 9700 RB, the Netherlands; 7 Department of Public Health, Erasmus MC, PO box 2040, Rotterdam 3000 CA, the Netherlands

**Keywords:** Chronic obstructive pulmonary disease (COPD), Emphysema, Smoking, Pulmonary function testing

## Abstract

**Background:**

Little is known about the factors associated with CT-quantified emphysema progression in heavy smokers. The objective of this study was to investigate the effect of length of smoking cessation and clinical / demographical factors on the rate of emphysema progression and FEV_1_-decline in male heavy smokers.

**Methods:**

3,670 male smokers with mean (SD) 40.8 (17.9) packyears underwent chest CT scans and pulmonary function tests at baseline and after 1 and 3 years follow-up. Smoking status (quitted ≥5, ≥1-<5, <1 years or current smoker) was noted. Rate of progression of emphysema and FEV_1_-decline after follow-up were assessed by analysis of variance adjusting for age, height, baseline pulmonary function and emphysema severity, packyears, years in study and respiratory symptoms. The quitted ≥5 group was used as reference.

**Results:**

Median (Q1-Q3) emphysema severity,<-950 HU, was 8.8 (5.1 – 14.1) and mean (SD) FEV_1_ was 3.4 (0.73) L or 98.5 (18.5) % of predicted. The group quitted ‘>5 years’ showed significantly lower rates of progression of emphysema compared to current smokers, 1.07% and 1.12% per year, respectively (p<0.001). Current smokers had a yearly FEV_1_-decline of 69 ml, while subjects quit smoking >5 years had a yearly decline of 57.5 ml (p<0.001).

**Conclusion:**

Quit smoking >5 years significantly slows the rate of emphysema progression and lung function decline.

**Trial registration:**

Registered at http://www.trialregister.nl with trial number ISRCTN63545820.

## Background

Chronic obstructive pulmonary disease (COPD) is a common pulmonary disease with an estimated prevalence of 8.9% worldwide [1]. Currently it is already one of the major causes of mortality and will become the third cause by 2020 [2]. COPD hallmarks are the accelerated lung function decline and emphysema progression. It is known that smoking cessation results in diminishing, but not in a total disappearance of inflammation and in dampening of the accelerated lung function decline [3-5]. Such is also expected for the emphysema progression over time, however few longitudinal studies in smokers have been performed studying emphysema progression [6].

Regarding emphysema progression in smokers, Suki et al. launched the hypothesis that the smoking related inflammation is not the only culprit, and that mechanical factors also play a role [7]. These mechanical factors are believed to be the start of an unstoppable cascade of increasing tissue destruction. A recent editorial addressed this as an important step in the understanding of emphysema progression [8]. Therefore, if inflammation is the only cause of emphysema progression this should be a function of the length of smoking cessation: none to minimal in subjects who stopped smoking a long time ago and strong in current smokers.

At a cross-sectional level it has been reported that CT-quantified emphysema is associated with demographical and clinical factors and an increasing number of studies report on effects of smoking habits on CT-quantified emphysema at a cross-sectional level [9-11]. However, there is little knowledge on the demographical and clinical factors associated with emphysema progression in heavy, but relatively healthy smokers.

The Dutch-Belgium lung cancer screening trial included a large number of relatively healthy, but heavy current and former smoking subjects, at risk for developing COPD [12]. A considerable fraction of these smokers stopped their habit and in this cohort we investigated the effect of the length of smoking cessation and secondarily some clinical / demographical factors on emphysema progression and lung function decline.

## Methods

### Participants

The included subjects were part of a lung cancer screening trial, the NELSON trial [12,13]. Male subjects, meeting the inclusion criterion of having smoked ≥20 packyears, were randomly sampled from the general population. Subjects should not have quit smoking more than 10 years before inclusion. Baseline details on smoking habits were gathered through questionnaires which included questions about duration of smoking, number of cigarettes smoked a day and, if applicable, the duration of smoking cessation at enrolment of the study (≥5 years, ≥1 - <5 years, <1 year and current smoker). Self-reported respiratory symptoms (cough, mucus, dyspnea and wheezing) were also collected via questionnaires at baseline.

**Figure 1:**
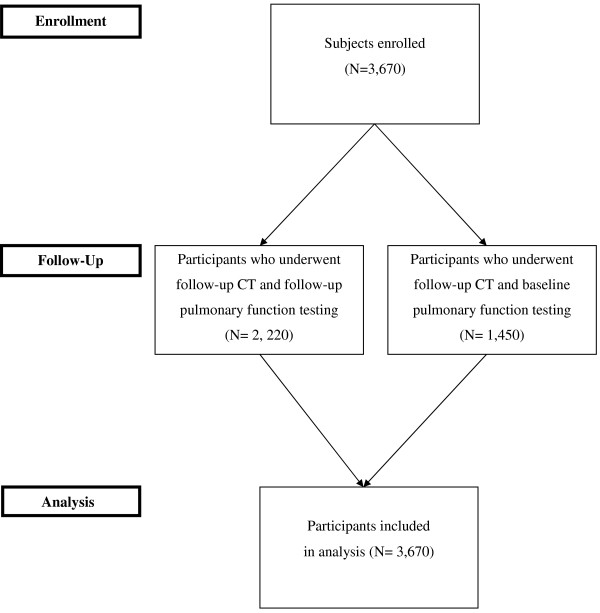
Flowchart of included subjects.

A chest CT scan was performed at least three times in all subjects: year 0 (at start study), year 1 and year 3. In addition, in a number of subjects ‘in between’ scans were made for follow-up of suspect nodules to exclude growth of these nodules which were also included. All included subjects underwent pulmonary function testing (PFT) at baseline. A random sample of approximately two out of three also underwent follow-up (PFT), see Figure 1.

The NELSON trial was approved by the Dutch Ministry of Health and by the institutional review boards of the participating centres. The NELSON trial is registered at http://www.trialregister.nl (trial number ISRCTN63545820). Written informed consent was obtained from all subjects.

### CT Scanning and quantification of emphysema

The CT protocol has been described in detail before and a more detailed description is also reported in the Additional file 1[13-15]. In short, all participants received low-dose CT during full inspiration. No spirometric gating was applied. There were no significant differences in CT derived inspiratory volume between the baseline and follow-up scans. Exposure settings were 30mAs at 120kVp or 140kVp. This low-dose CT protocol has previously been used to quantify emphysema in COPD patients and heavy smokers [12-14]. All CT scans were automatically analyzed [16]. CT examinations were recalibrated using air in the trachea to ensure comparability between the two centers. Quality control was done by scanning a phantom before and after scan sessions. The phantom was scanned before and after each date acquisition session to see if the range of HU values was in the range of that specified by the vendor and to determine systematic deviations. During the trial no systematic deviations in HU value occurred. Emphysema quantification was based on the -950 HU and -910 HU technique: this method delivers the percentage lung volume with a density <-950 HU or <-910 HU. As secondary parameters the 15th percentile technique was used: providing the cut off value in Hounsfield units (HU) below which 15% of all voxels are distributed [17].

### Pulmonary function tests

Pulmonary function tests (PFT) were performed in year 0 and 3 with standardized equipment according to American Thoracic Society guidelines including FEV_1_ and FEV_1_/ forced vital capacity (FVC) [18]. Bronchodilatation was not applied. Baseline FEV_1_/FVC and FEV_1_ served to assess whether airflow obstruction was present (if FEV_1_/FVC <70%) and to determine the baseline GOLD stage [19].

### Statistical analysis

Mean and standard deviation (SD) values were calculated for normally distributed data and median values and inter quartile range (Q1-Q3) for non-normally distributed data. Distribution of normality was checked visually by Q-Q plots.

Baseline data were evaluated using analysis of variance or χ [2]-test, where appropriate. For these longitudinal data a linear mixed model (a random intercept, random slope variant with an unstructured covariance matrix) was used. Linear mixed modeling results in a linear regression equation with (amongst others) time as an explanatory parameter, while correcting for the correlation between observations. The period of observation since the start of the study per subject was the time factor in this analysis.

The variable of interest was the ‘percentage lung volume <-950 HU’ and as this is a log-normal distributed parameter we applied a log (ln) transformation first. Smoking status at enrolment of the study (quitted ≥5 years, ≥1 - <5 years, <1 year or current smoker) was the main explanatory factor. To assess whether the progression of emphysema differed between these smoking groups, the interaction between the smoking status and ‘time of observation’ was the next relevant factor. The quitted ≥5 years group was used as a reference. We adjusted for presence of respiratory symptoms (cough, wheezing, dyspnea and /or mucus), packyears of smoking, baseline GOLD stage, study center, height and age at start study. The FEV_1_ decline was analyzed in the same way as the emphysema progression. P-values ≤0.05 were considered significant. Statistical analyses were performed via SPSS20 (Chicago, USA).

## Results

### Baseline demographics and lung function results

Baseline demographics and lung function test outcomes are shown in Table 1. In total 3,670 male subjects of middle age (~60 years) were included. All were current (n= 1,614) or former heavy (n=2,056) smokers with a mean (SD) packyears of 40.8 (17.9). The majority of these subjects, 2,403 (65%), had an FEV_1_/FVC >70%, but there were no differences in airflow obstruction prevalence between the four smoking groups (p= 0.233). Also no significant differences in FEV_1_ and FEV_1_/FVC were present between the four smoking groups (p= 0.415 and p=0.185, respectively). Of the 3,670 subjects included a random sample of 2,220 also underwent follow-up pulmonary function testing, see Figure 1. Current smokers significantly had more respiratory complaints (Table 1).

**Table 1:** Baseline demographics for the total cohort and split by smoking status

	***Total n = 3,670***	***Current smoker n= 2,056***	***Quitted <1 year n=284***	***Quitted ≥1 – <5 years n=711***	***Quitted ≥5years n=619***
**Age**	59.8 (5.4)	58.9 (4.9)	59.8 (5.3)	60.4 (5.5)	62.1 (6.2)
**height (cm)**	178 (7)	178 (7)	179 (7)	178 (7)	177 (7)
**FEV**_**1**_**/FVC**	72.2 (9.4)	72.8 (9)	72.8 (9.1)	71.8 (10.2)	71.9 (9.4)
**FEV**_**1 **_**[L]**	3.4 (0.73)	3.4 (0.8)	3.4 (0.7)	3.5 (0.8)	3.4 (0.7)
**FEV**_**1 **_**[%]**	98.5 (18.5)	99.9 (20)	100 (18)	97.1 (19.4)	97.6 (17.9)
**Packyears**	40.8 (17.9)	40.0 (16.4)	41.0 (17.5)	43.1 (20.2)	40.6 (19.5)
observation time (years)*	3.0 (2.9 – 3.1)	3.0 (2.9-3.1)	3.0 (2.9-3.1)	3.0 (2.9-3.1)	3.0 (2.9-3.1)
**GOLD classification**					
normal (%)	65.5	63.2	65.5	68.8	69.1
stage I (%)	22.0	23.4	20.4	20.8	19.2
stage II (%)	10.9	11.6	12.0	9.1	10.3
stage III (%)	1.6	1.8	2.1	1.3	1.5
stage IV (%)	0.1	0.1	0	0	0
**Presence of respiratory symptoms**					
cough (%)	32.0	46.0	24.9	14.5	10.7
wheezing (%)	23.8	33.0	20.9	13.1	7.4
dyspnea (%)	29.5	34.4	29.5	23.3	20.8
mucus (%)	30.0	39.6	26.0	17.8	15.2
lung volume <-950 HU (%)*	8.8 (5.1 – 14.1)	6.8 (3.9 – 11.5)	10.5 (6.7 – 15.2)	11.3 (7.4 – 17.2)	11.9 (7.5 – 16.9)
lung volume <-910 HU (%)*	35.2 (24.1 – 46.0)	30.2 (20.1 – 41.9)	38.4 (28.7 – 47.9)	40.9 (30.6 – 50.7)	41.2 (31.6 – 51.4)
**perc15 (HU)**	−935 (19)	−930 (21)	−939 (16)	−942 (18)	−942 (17)

Mean and standard deviation (SD) is provided*median (Q_25_ – Q_75_).

### Baseline emphysema

The effect of the significant parameters on % lung volume <-950 HU are given in Table 2. Current smokers had less emphysema at baseline compared to the long term quitters. This contra-intuitive phenomenon has been described earlier and is believed to mirror the presence of inflammation in smokers, which thickens the mucosa and increases tissue density [20]. Subjects with the lowest GOLD stage showed less emphysema severity compared to those with the highest GOLD stage (p=0.013). Respiratory symptoms had no significant effect on baseline emphysema. Similar results were found for the <-910 HU and for the Perc15 (Additional file 1: Table S1).

**Table 2:** Effect estimates of listed parameters on baseline % lung volume <-950 HU values

***Parameter***	***Effect size***	***p-value.***	***95% CI of effect size***	
			***Lower bound***	***Upper bound***
**Center**				
UMCU	0.87	<0.001	0.83	0.91
UMCG (reference)				
**smoking group**				
current smoker	0.60	<0.001	0.56	0.64
quitted <1 year	0.90	0.033	0.81	0.99
quitted ≥1 – <5 years	0.99	0.956	0.93	0.99
quitted ≥5years (reference)				
**baseline GOLD stage**				
normal	0.17	0.013	0.04	0.68
stage I	0.26	0.065	0.06	1.08
stage II	0.29	0.089	0.07	1.21
stage III	0.46	0.279	0.11	1.89
stage IV (reference)				
**Presence of respiratory symptoms**				
cough	0.87	0.980	0.82	0.91
wheezing	0.97	0.449	0.90	1.05
dyspnea	1.00	0.786	0.94	1.08
mucus	1.02	0.568	0.95	1.09
**height (cm)**	1.01	<0.001	1.01	1.02
**age at start study (years)**	1.02	<0.001	1.01	1.02
**packyears**	0.99	<0.001	0.99	1.00

The column headed with ‘effect size’ gives the change in *% lung volume <-950 HU* due to a unit change in the parameter or due to membership of another class. So for each year a subject is older at the start of the study, the *% lung volume <-950 HU* increases with a factor or1.02 per year. For class comparisons the changes are versus the reference group: so a subject with an FEV1/FVC >70% (no COPD) has 0.17 times lower *% lung volume <-950 HU* compared to the reference GOLD class 4 (the p-value for this comparison is <0.001).

### Effects on rate of emphysema progression

The median (Q1-Q3) time of observation was 3.0 (2.9 – 3.1) years. All factors in the model significantly influenced (all p<0.001) the severity of emphysema, except for study center (p=0.619).

Table 3 lists the results from the linear mixed model. The annual emphysema increase for all subjects (irrespective of smoking status) was highly significant (p<0.001) and was 1.07% of the total lung volume per year. For instance, a subject with a -950 HU value of 8.8%, being the mean value of the cohort, will show a -950 HU value of 9.1% after three years.

**Table 3:** Effect estimates of listed parameters on the increase of the % lung volume <-950 HU values

***Parameter***	***Effect size***	***p-value.***	***95% CI of effect size***	
			***Lower bound***	***Upper bound***
**center**				
UMCU	1.01	0.619	0.97	1.05
UMCG (reference)				
**smoking group**				
current smoker	0.60	<0.001	0.56	0.64
quitted <1 year	0.91	0.083	0.82	1.01
quitted ≥1 – <5 years	0.99	0.868	0.92	1.07
quitted ≥5years (reference)				
**baseline GOLD stage**				
normal	0.18	0.002	0.06	0.54
stage I	0.28	0.022	0.09	0.83
stage II	0.31	0.040	0.10	0.95
stage III	0.47	0.183	0.15	1.43
stage IV (reference)				
**height (cm)**	1.01	<0.001	1.07	1.01
**Presence of respiratory symptoms**				
cough	0.96	0.647	0.93	1.05
wheezing	1.01	0.881	0.94	1.07
dyspnea	0.99	0.773	0.94	1.05
mucus	1.02	0.724	0.96	1.09
**age at start study (years)**	1.02	<0.001	1.01	1.02
**packyears**	0.99	<0.001	0.97	0.99
**observation time**	1.07	<0.001	1.06	1.09
observation time * smoking group				
current smoker	1.05	<0.001	1.03	1.07
quitted <1 year	0.99	0.799	0.97	1.02
quitted ≥1 – <5 years	0.99	0.234	0.97	1.01
quitted ≥5years (reference)				

The column headed with ‘effect size’ again contains the change in *% lung volume <-950 HU* due to a unit change in the parameter or due to membership of another class. For instance, for each year a subject is older at the start of the study, the *% lung volume <-950 HU* increases with a factor of 1.02. For class comparisons the changes are versus the reference group: so a subject with an FEV1/FVC >70% (no COPD) has a 0.18 times lower *lung volume <-950 HU* compared to the reference GOLD class 4 (the p-value for this comparison is 0.002). The parameter *observation time* denotes the annual change in *% lung volume <-950 HU* being times 1.07 higher per year.

**Figure 2:**
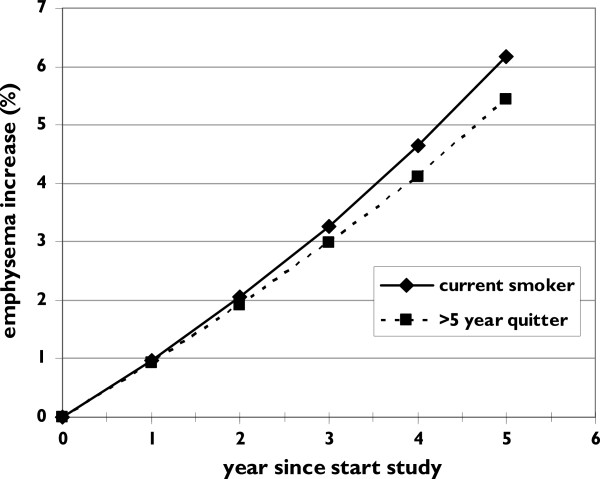
**Graph showing the increase of emphysema (% lung volume below -950 HU) in two men, age 60 at the start of the observation (t=0) with 40 packyears smoked in GOLD stage 1 (height 175 cm).** One is a current smoker, the other a >5 year quitter. For sake of clarity the other two smoking groups were omitted from this graph as their emphysema increase is within these extreme groups.

The interaction between ‘smoking group’ and ‘observation time’ indicates that the development of emphysema differed between these groups (p<0.001). Only the current smokers differed from the reference group (= ≥5 years quitters) (p<0.001): the additional yearly increase per year is 1.05 (see Table 3, observation time * smoking group). So a current smoker faces a total emphysema increase of 1.12% per year, see Figure 2. The analysis could not show that the <1 year and ≥1 - <5 years quitters differed significantly from the ≥5 years quitters in additional decline (p=0.799 and p=0.234): these two groups seem to suffer from a similar yearly emphysema increase. The effect of aging was estimated as a 1.02% increase per year: for each year a subject gets older, the %<-950 increases with 1.02%. Table 3 lists the results from the linear mixed model. No association with respiratory symptoms was found. Similar results were found for <-910 HU and Perc15 (Additional file 1).

### Effects on FEV_1_-decline

**Figure 3:**
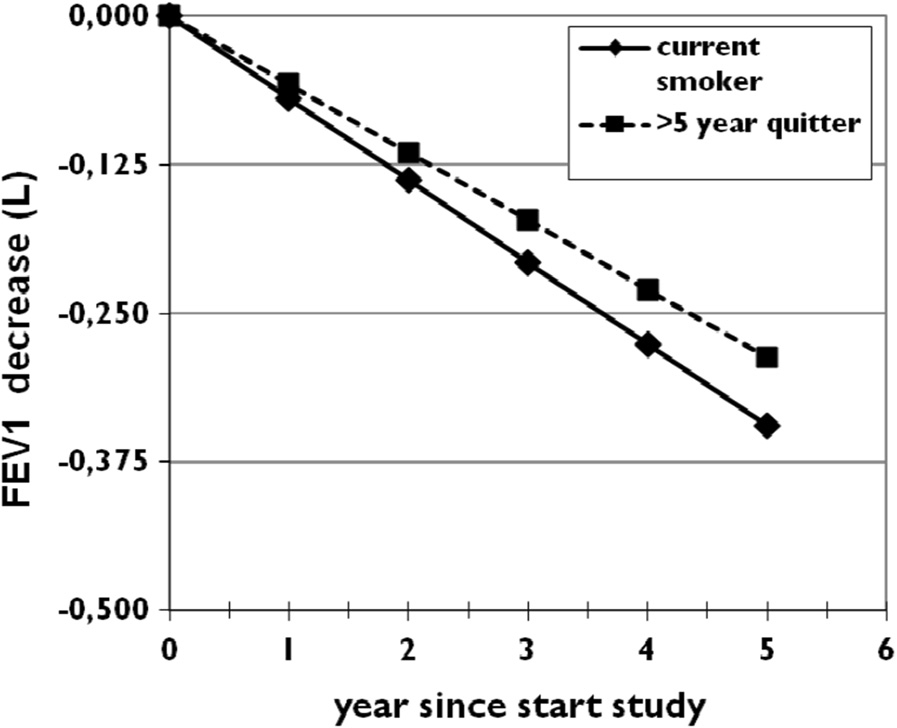
**Graph showing the decrease of FEV**_**1 **_**in two men, age 60 at the start of the observation (t=0) with 40 packyears smoked in GOLD stage 1 (height 175 cm).** One is a current smoker, the other a >5 year quitter. For sake of clarity the other two smoking groups were omitted from this graph as their FEV_1_ decrease.

The mixed model analysis showed that all investigated factors significantly influenced the level of FEV_1_-decline (all p<0.001). The annual FEV_1_ decline for all subjects (irrespective of smoking status) was 57.5 ml/year (p<0.001, see Table 4). The interaction between ‘smoking group’ and the ‘observation time’ was highly significant (p<0.001) indicating that the FEV_1_ decline differed between the groups. Current smokers differed significantly from the reference group (= ≥5 years quitters), with an additional yearly decline per of 11.5 ml (p<0.029). So current smokers faced a total FEV_1_ decline of 69 ml/ year (57.5 ml + 11.5 ml), see Figure 3. The analysis furthermore showed that the <1 year quitters differed significantly from the ≥5 years quitters in additional decline (p=0.019), but not from the ≥1 - <5 years quitters (p=0.237). The effect of normal aging is estimated to be a decrease of 29 ml per year [21].

**Table 4:** **Effect estimates of listed parameters on the longitudinal FEV**_**1 **_**(milliliters) values**

***Parameter***	***Effect size***	***p-value.***	***95% CI of effect size***	
			***Lower bound***	***Upper bound***
**center**				
UMCU	-.74.4	<0.001	−103	46
UMCG (reference)				
**smoking group**				
current smoker	−101	<0.001	−142	−61
quitted <1 year	−68	0.033	−130	−5
quitted ≥1 – <5 years	−32	0.188	−80	15
quitted ≥5years (reference)				
**baseline GOLD stage**				
normal	2346	<0.001	1497	3195
stage I	2035	<0.001	1185	2884
stage II	1187	0.006	337	2037
stage III	346	0.428	−510	1202
stage IV (reference)				
**height (cm)**	044	<0.001	41	46
**Presence of respiratory symptoms**				
cough	−11	0.607	−7	19
wheezing	−106	<0.001	−152	−60
dyspnea	−76	<0.001	−117	−34.7
mucus	12	0.580	−30	54
**age at start study (years)**	−29	<0.001	−32	−27
**packyears**	−3.2	<0.001	−4	−2
**observation time**	−58	<0.001	−66	−48
observation time * smoking group				
current smoker	−12	0.029	−22	−1
quitted <1 year	−19	0.019	−35	−3
quitted ≥1 – <5 years	0.8	0.237	−5	20
quitted ≥5years (reference)				

The column headed with ‘effect size’ contains the change in FEV1 *(millliters)* due to a unit change in the parameter or due to membership of another class. So for each year a subject is older at the start of the study the FEV1 decreases with 29 millliters.

### Correlation between emphysema progression and lung function decline

The FEV_1_-decline was not significantly correlated to the emphysema progression over the observation time (R= 0.004675; p= 0.826).

## Discussion

The results show that in this large cohort of heavy current and former smokers, the progression of emphysema was lower in >5 years quitters compared to current smokers. The FEV_1_ decline reduced with increasingly longer stopped smoking duration. However, even in long-term quitters the lung function decline was higher as expected compared to normal lung function decline. Although we found a significant effect of smoking cessation on emphysema progression, the size of the effect was not as high as we expected or hoped for. Our data thus seem to confirm the hypothesis of Suki et al who proposed that the initial inflammation is believed to permanently damage / weaken the collagen fibers with subsequent mechanical failure [7]. It could well be that mechanical factors play an important role in starting an unstoppable cascade of tissue destruction not stopped by long term smoking cessation resulting in ongoing progression of emphysema in heavy smokers. Indeed, al included participants were heavy smokers with mean packyears smoked of 40 years.

The effect of smoking cessation on emphysema progression and lung function decline differ, i.e. there was a larger effect on lung function. It may be that reduction of emphysema progression takes more time. Smoking related inflammation should have dampened during our long non-smoking follow-up period of 5 years. After smoking cessation, the pathological processes leading to the accelerated FEV_1_-decline need some time to diminish. A large study in male UK doctors reported it took ~1 year before all COPD-related mortality rates declined [22]. Two cross-sectional studies found a decrease in the blood and bronchoalveolar lavage fluid leukocyte levels after nine months of smoking cessation, when compared to never smokers [23,24]. Together, these results indicate that the increased inflammation should be halted during the 5 years non-smoking period of the former smokers.

Only a few other studies longitudinally examined the effect of smoking status on emphysema progression. Bellomi *et al.* found that, after 2 year of follow-up, current smokers showed significantly stronger emphysema progression than former smokers [6]. The design of the study was comparable to ours: subjects participated in a lung cancer screening trial and underwent low-dose CT. However, no lung function was obtained and it may not be unlikely that significant differences in FEV_1_/FVC and FEV_1_ values existed between the former and current smokers. Furthermore, they did not take the length of smoking cessation into account. Soejima *et al.* found no significant differences in emphysema progression between former and current smokers after a five year follow-up [25]. A drawback is that the number of subjects included was very small, 35 current smokers and 12 past smokers, which could have lead to a lack of power. A recent study by Coxson et al. is of special interest as they also studied emphysema progression assessed by CT, but in a more severe population as ours [26]. They studied 1,928 male and female subjects with COPD stage II-IV and found that emphysema progression was more severe in current smokers and in females. These findings are in line with our findings, but are complementary as we included heavy smokers without or only mild COPD.

Our results expand / confirm the outcome of e.g. the Lung Health Study (LHS), a study investigating the effect of smoking cessation in COPD subjects which found that quitters had a considerably lower FEV_1_ decline than continuous smokers [27]. The average yearly FEV_1_ decline was 62 ml in continuous smokers, and 31 ml in quitters. In our cohort this was slightly higher, namely 69 and 57.5 ml/ year, respectively. There are some important differences between our study and the LHS. Firstly, the LHS included subjects with COPD, while the vast majority of our subjects had no COPD. The population studied in the LHS was relatively young, with a mean age below 50 years, but with the same exposure to tobacco as our population (~40 packyears). The LHS thus included younger smokers with an apparent increased COPD susceptibility.

As expected we found the tissue density in ex-smokers to be lower compared to current smokers [20]. This is probably caused by the presence of inflammation and mucus which influences emphysema quantification by raising the density of the lungs. In the long-term quitters inflammation and mucus production will have disappeared before the observation period started and changes over time cannot be influenced by it. In current smokers the inflammation and mucus production increased density, but as long as this is constant it is not an influencing factor for changes over time. A further increase in density (via an increased smoking burden) would again reduce the emphysema progression, as found on CT. Another explanation could be the so-called healthy smoker effect in which smokers with little respiratory complaints keep smoking while subjects who have respiratory complaints stop at an early stage.

A positive point of our study is that we included a large sample of heavy smoking subjects selected from the general population and at risk to the develop COPD, rendering our results applicable to the vast number of so-called ‘healthy smokers’. Especially this group is of interest because smoking cessation is the most effective intervention and may prevent progression to COPD. Secondly, emphysema was quantified fully automatic by soft-ware packages, free from inter- and intra-observer variability.

This study also has some limitations. Firstly, smoking cessation was self-reported. Some subjects could have restarted smoking during the observation period; however this only would dilute the reported effects of the length of smoking cessation. So we expect to underestimate true effects. Secondly, no females were included in our study: previous research showed that females benefit more from smoking cessation which may imply that women may have less emphysema progression after smoking cessation [28,29]. It is not expected that emphysema progression will be less in women, but it at the least will be similar or larger compared to men. Female sex may also be a risk factor for an increased emphysema progression [26,30]. Future studies should include females to elucidate this. It has been reported that inspirational levels affect the quantification of emphysema at CT. Thirdly, one of the limitations of the quantitative analysis of low attenuation voxels at CT is that other conditions, like for instance mucus in the airways or atelectasis, are not incorporated in the single emphysema measurement. To our knowledge, this is inevitable with the current soft-ware and quantification methods. Ideally, one may spirometrically gate the CT scans to ensure that repeat CT scans are acquired at the same inspirational level. However, this is not feasible in large multi-center studies. In our study we inserted all the individual inspirational levels in the analyses and found that they did not influence the outcomes (data not reported). A last issue might be that we cannot assess the effects of the increasing emphysema severity on e.g. diffusing capacity and static lung volumes, as these were not part of this longitudinal study.

In conclusion, we have showed that smoking cessation for ≥5 years decreased lung function decline when compared with current smokers. However, the effect was not as high as expected giving support to the hypothesis that long term smoking starts a unstoppable cascade of tissue damaging. Nonetheless, our results emphasize on the benefits of smoking cessation, regardless of being a heavy smoker.

## Abbreviations

COPD: Chronic obstructive pulmonary disease; CT: Computed tomography; FEV1: Forced expiratory volume in one second; FVC: Forced vital capacity; GOLD: Global Initiative for Chronic Obstructive Lung Disease; HU: Hounsfield unit; LLN: Lower limit of normal; LHS: Lung Health Study; MEF5: Maximum expiratory flow at 50% of FVC; mL: Milliliter; NELSON: Dutch abbreviation for Dutch-Belgian Lung Cancer Screening Trial Perc15, CT derived emphysema severity measure representing the Hounsfield units (HU) point below which 15% of all voxels are distributed.; PFT: Pulmonary function tests; Q1-Q3: 25th – 75th percentile; SD: Standard deviation.

## Competing interests

DS Postma received funding for research from AstraZeneca, GSK, Nycomed. Travel to ERS or ATS has been partially funded by AstraZeneca, GSK, Chiesi, Nycomed. DS Postma has been consultant to AstraZeneca, Boehringer Ingelheim, Chiesi, GSK, Nycomed, TEVA. The other authors state that they have no conflicts of interest.

## Authors’ contribution

FMH and PZ were responsible for the concept and design of the study and the statistical analyses and are guarantor of the paper. BG was responsible for the quantification of emphysema. HB, HG, DP,JL, HK, MO and PJ assisted in designing the study and writing of the manuscript. All authors contributed to writing and all approved the final version of the manuscript.

## Supplementary Material

Additional file 1 Results when using Perc15 and %<-910 as emphysema measurement. Click here for file
