# Supplementary material for: Rate of progression of CT-quantified emphysema in male current and ex-smokers: a follow-up study
Source: Respir Res. 2013 May 20;14(1):55. doi: 10.1186/1465-9921-14-55 (PMC3669040; doi:10.1186/1465-9921-14-55)
Supplement: Additional file 1 — Results when using Perc15 and %<-910 as emphysema measurement. [file 1465-9921-14-55-S1.doc]

**Supplementary files**

**Rate of progression of CT-quantified emphysema in current**

**and ex-smokers: a follow-up study**

**Methods**

**CT scanning**

All participants received low-dose CT without intravenous contrast injection. At both screening sites 16-detector MDCT scanners were used (Brilliance 16P, Philips Medical Systems, Cleveland, OH, USA or Sensation-16 Siemens Medical Solutions, Forchheim, Germany). CT scans were obtained in spiral mode, with 16 x 0.75mm collimation and in full inspiration. No spirometric gating was applied since it has been reported that this does not improve repeatability of lung density measurements. Axial images were reconstructed with 1.0mm thickness at 0.7mm increment. All CT scans were reconstructed with a soft reconstruction filter (Philips B, Siemens B30f) at a 512x512 matrix. Exposure settings were 30mAs at 120kVp or 140kVp, depending on participant’s weight, ≤80 and >80 kilograms, respectively. This low-dose CT protocol was applied in order to reduce the risk of inducing a neoplasm due to radiation.

**Results
Table S1 Effect estimates of listed parameters on baseline ln_transformed % lung volume <-910 HU values. For an explanation see table 2.**

| ***Parameter*** | ***effect size*** | ***p-value.*** | ***95% CI of effect size*** | |
| --- | --- | --- | --- | --- |
|  |  |  | ***lower bound*** | ***upper bound*** |
| **Center** |  |  |  |  |
| UMCU | -0.181 | <0.001 | -0.149 | 0.213 |
| UMCG (reference) |  |  |  |  |
| **smoking group** |  |  |  |  |
| current smoker | 0.295 | <0.001 | 0.250 | 0.340 |
| quitted <1 year | 0.048 | 0.174 | -0.021 | 0.117 |
| quitted ≥1 – <5 years | -0.004 | 0.870 | -0.057 | 0.049 |
| quitted ≥5years (reference) |  |  |  |  |
| **baseline GOLD stage** |  |  |  |  |
| normal | 0.879 | 0.071 | -0.077 | 1.835 |
| stage I | 0.605 | 0.215 | -0.351 | 1.561 |
| stage II | 0.597 | 0.221 | -0.360 | 1.554 |
| stage III | 0.365 | 0.458 | -0.599 | 1.328 |
| stage IV (reference) |  |  |  |  |
| **Presence of respiratory symptoms** |  |  |  |  |
| cough | -0.030 | 0.229 | -0.078 | 0.019 |
| wheezing | -0.039 | 0.133 | -0.090 | 0.012 |
| dyspnea | -0.032 | 0.177 | -0.077 | 0.014 |
| mucus | 0.029 | 0.228 | -0.018 | 0.075 |
| **height (cm)** | 0.009 | <0.001 | 0.006 | 0.011 |
| **age at start study (years)** | 0.011 | <0.001 | 0.008 | 0.014 |
| **packyears** | -0.002 | <0.001 | -0.003 | -0.002 |

**Table S2** *Effect estimates of listed parameters on the longitudinal Perc15 values.*

| ***parameter*** | ***effect size*** | ***p-value.*** | ***95% CI of effect size*** | |
| --- | --- | --- | --- | --- |
|  |  |  | ***lower bound*** | ***upper bound*** |
| **center** |  |  |  |  |
| UMCU | 0.010 | .619 | -0.030 | 0.051 |
| UMCG (reference) |  |  |  |  |
| **smoking group** |  |  |  |  |
| current smoker | -0.507 | <0.001 | -0.573 | -0.441 |
| quitted <1 year | -0.089 | 0.083 | -0.191 | 0.012 |
| quitted ≥1 – <5 years | -0.007 | 0.868 | -0.084 | 0.071 |
| quitted ≥5years (reference) |  |  |  |  |
| **baseline GOLD stage** |  |  |  |  |
| normal | -1.721 | 0.002 | -2.822 | -0.620 |
| stage I | -1.287 | 0.022 | -2.389 | -0.185 |
| stage II | -1.156 | 0.040 | -2.259 | -0.053 |
| stage III | -0.756 | 0.183 | -1.868 | 0.356 |
| stage IV (reference) |  |  |  |  |
| **height (cm)** | 0.009 | <0.001 | 0.006 | 0.012 |
| **age at start study (years)** | 0.016 | <0.001 | 0.012 | 0.020 |
| **packyears** | -0.002 | <0.001 | -0.003 | -0.001 |
| **observation time** | 0.069 | <0.001 | 0.055 | 0.083 |
| **observation time * smoking group** |  |  |  |  |
| current smoker | 0.048 | <0.001 | 0.032 | 0.064 |
| quitted <1 year | -0.003 | 0.799 | -0.028 | 0.022 |
| quitted ≥1 – <5 years | -0.012 | 0.234 | -0.031 | 0.008 |
| quitted ≥5years (reference) |  |  |  |  |

**Table S3** Effect estimates of listed parameters on the longitudinal ln_transformed % lung volume <-910 HU values.

| ***parameter*** | ***effect size*** | ***p-value.*** | ***95% CI of effect size*** | |
| --- | --- | --- | --- | --- |
|  |  |  | ***lower bound*** | ***upper bound*** |
| **center** |  |  |  |  |
| UMCU | 0.209 | <0.001 | 0.181 | 0.236 |
| UMCG (reference) |  |  |  |  |
| **smoking group** |  |  |  |  |
| current smoker | 0.269 | <0.001 | 0.230 | 0.308 |
| quitted <1 year | 0.049 | 0.113 | -0.012 | 0.109 |
| quitted ≥1 – <5 years | 0.010 | 0.676 | -0.036 | 0.056 |
| quitted ≥5years (reference) |  |  |  |  |
| **baseline GOLD stage** |  |  |  |  |
| normal | 0.870 | 0.023 | 0.121 | 1.618 |
| stage I | 0.608 | 0.111 | -0.141 | 1.357 |
| stage II | 0.580 | 0.130 | -0.170 | 1.329 |
| stage III | 0.378 | 0.328 | -0.379 | 1.134 |
| stage IV (reference) |  |  |  |  |
| **height (cm)** | 0.007 | <0.001 | 0.005 | 0.009 |
| **Presence of respiratory symptoms** |  |  |  |  |
| cough | -0.030 | 0.162 | -0.071 | 0.012 |
| wheezing | -0.012 | 0.604 | -0.056 | 0.032 |
| dyspnea | -0.042 | 0.037 | -0.081 | -0.002 |
| mucus | 0.023 | 0.259 | -0.017 | 0.063 |
| **age at start study (years)** | 0.009 | <0.001 | 0.006 | 0.011 |
| **packyears** | -0.002 | <0.001 | -0.003 | -0.001 |
| **observation time** | 0.009 | 0.058 | -0.001 | 0.018 |
| **observation time * smoking group** |  |  |  |  |
| current smoker | 0.027 | <0.001 | 0.016 | 0.038 |
| quitted <1 year | -0.002 | 0.843 | -0.018 | 0.015 |
| quitted ≥1 – <5 years | -0.009 | 0.147 | -0.022 | 0.003 |
| quitted ≥5years (reference) |  |  |  |  |
